# Spatial Variation in EU Poverty with Respect to Health, Education and Living Standards

**DOI:** 10.1007/s11205-014-0848-7

**Published:** 2014-12-12

**Authors:** Dorota Weziak-Bialowolska

**Affiliations:** Econometrics and Applied Statistics Unit, Deputy Directorate General, Joint Research Centre, European Commission, Via E. Fermi 2749, TP 361, 21027 Ispra, VA Italy

**Keywords:** Degree of urbanisation, European Union, EU-SILC, Multidimensional poverty

## Abstract

We examine the European Union (EU) countries and within-country areas (i.e., large urban areas, small urban areas, and rural areas) that are the most disadvantageous with respect to multidimensional poverty and in each of the investigated dimensions, i.e., health, education, and living standards. To this end, we construct the Multidimensional Poverty Index and its sub-indices: the Poverty in Health Index, Poverty in Education Index, and Poverty in Standard of Living Index. All of these indices provide information regarding the fraction of people who live in poverty, as well as information on the poverty intensity experienced by the poor. Our results indicate that the scale of poverty in the EU countries is diversified, with Denmark and Sweden being the most affluent countries, and Latvia, Bulgaria, and Romania being the most disadvantageous. We demonstrate that there are countries with no differences in the levels of poverty within a country, such as Denmark, Sweden, Spain, Finland, and the Czech Republic, and countries, usually less affluent ones such as Romania, Bulgaria and Lithuania, where considerable geographical inequality is present. In general, in countries with a high and moderately high number of poor, the worst situation with respect to the scale of poverty is observed in rural areas, and the best situation is observed in large urban areas, with the exception of Greece, Italy, and Portugal, where in large urban areas, the situation is the worst. In countries with a low number of poor, in general, the poverty is relatively higher in large urban areas.

## Introduction

The standard of people’s lives, both in relative terms (compared to other people in society) and in absolute terms (whether they enjoy the basic necessities of life), is a reflection of whether people live in poverty. However, the notion of poverty is understood and operationalised differently in different contexts (Callander et al. 2012), and accordingly, its types are also categorised differently.

According to Wagle (2008) and Saunders (2005), there are three main approaches to the conceptualisation and operationalisation of poverty: economic well-being, capability (which stems from the economic well-being approach) and social inclusion. The economic well-being concept links poverty to the economic deprivation that, in turn, relates to material aspects and/or standards of living. The capability approach proposed by Sen (1993) expands the notion of poverty from welfare, consumption and income to broader concepts such as freedom, well-being, and capabilities. In his approach, poverty is understood as a state of capability or functioning deprivation that happens when people lack freedom and opportunities to acquire or expand their abilities. The third approach, which is based on social exclusion, stems from the process of systematic isolation, rejection, humiliation, lack of social support, and denial of participation (Wagle 2008).

Depending on the type of definition, not only is poverty addressed by means of numerous dimensions, such as poverty in education, health, and living standards (Alkire and Foster 2011a, b; Antony and Visweswara Rao 2007; Betti et al. 2012; Weziak-Bialowolska and Dijkstra 2014), but different indicators are also selected for its measurement. Such indicators can be generally classified into income and nonincome related and are usually implemented in the multidimensional conceptualisation of poverty. Proponents of such an approach are, for example, Alkire and Foster (2011a, b), Alkire and Santos (2013), Annoni and Weziak-Bialowolska (2014), Antony and Visweswara Rao (2007), Bellani (2013), Betti et al. (2012), Callander et al. (2012), Dewilde (2004, 2008), Merz and Rathjen (2014), Ravallion (2011, 2012) and Wagle (2008), among others.

Researchers measuring poverty generally present their results by either referring to countries with a clear differentiation between developing (Alkire and Foster 2011b; Alkire and Santos 2010, 2013) and developed, usually the European Union (EU) countries (Atkinson et al. 2004; Dewilde and Vranken 2005; Dewilde 2008; Nolan and Whelan 2010), or to the country-specific populations experiencing it. For example, Miranti et al. (2011), and Tanton et al. aimed to compare the situations in urban and rural areas in Australia; Weber et al. (2005), in the United States (US). Jolliffe (2006) and Wang et al. (2011) explored poverty in the US metropolitan and nonmetropolitan areas; Kemeny and Storper (2012), within the US metropolitan areas; and Annoni and Weziak-Bialowolska (2014) and Longford et al. (2012), among the EU regions.

Although there are studies on multidimensional poverty in the EU countries (Dewilde and Vranken 2005; Dewilde 2004, 2008), it must be noted that they often rely on the data from the European Community Household Panel (ECHP) study, which means that, at best, they address the situation as it was in 2001. In addition, studies on multidimensional poverty distribution with respect to the different types of urbanisation areas in the EU countries are considerably limited. It is astonishing, first, because the comparisons of the situation in (1) rural areas, (2) towns and suburbs/small urban areas, and (3) cities or large urban areas, i.e., the categories of the degree of urbanisation classification, are not only natural and common but are also widely used in the field of poverty (and not only) by the general public, policymakers, researchers, national administrators, and international organisations such as the Organisation for Economic Cooperation and Development (OECD), the United Nations (UN), and the European Commission (EC) (Dijkstra and Poelman 2014). Second, the levels of poverty in rural and urban areas are not only different, as indicated by Priavin (1980) and Tanton et al. (2010), but the poverty observed there often has different causes, correlates and solutions (Atkinson et al. 2010; Wang et al. 2011). This implies that taking the degree of urbanisation as a braking variable in the research of the magnitudes of within-country and between-country poverty is meaningful.

We are aware that there is a measure of poverty officially used in the EU, namely, the ‘at risk of poverty or social exclusion’ (AROPE) rate.^1^ This measure refers to the situation of people either at risk of poverty, severely materially deprived or living in a household with a very low work intensity, which implies that it measures poverty from the multidimensional perspective. It is also reported for differently defined areas with respect to the degree of urbanisation. However, first, it does not take into account poverty in such fields as education and health. Second, it informs about the share of poor but does not take into account the depth and intensity of poverty.

Therefore, the aim of this paper is to determine the EU countries and within-country areas, defined with respect to the degree of urbanisation, that are the most disadvantageous with respect to multidimensional poverty in general and in each of the investigated dimensions, i.e., living standards, health, and education.^2^ To this end, we propose a composite indicator named the Multidimensional Poverty Index (MPI) that, first, adopts the economic well-being approach; second, it enables identification of the poor; third, it aggregates dimensions to obtain an overall measure of poverty reflecting the multiple deprivations experienced by the poor; fourth, it enables the assessment of not only the share of the poor but also the intensity of the poverty that is experienced.

To the best of our knowledge, our approach features the following innovative points. First, we focus on regional variability because not only are the EU countries diversified with respect to poverty levels, but also, local differences in poverty are essential for targeted antipoverty policies. Second, we use the European Union Survey on Income and Living Condition (EU-SILC) as a main data source, which enables us to provide comparable information about poverty in 24 EU countries.

In the following sections, we first present the conceptualisation of the measurement of poverty from the multidimensional and nonincome perspectives and the data sources. Next, we describe the methods applied to construct the MPI and to assess its robustness. The results obtained for 24 EU countries and 69 EU areas are presented in the following section, in which we present the geographical distribution of multidimensional poverty, poverty in health, poverty in education, and poverty in living standards in the EU and particular scenario solutions. Additionally, we investigate the criterion validity of the MPI by examining the association between the MPI and the AROPE rate. The final section is devoted to the presentation of the conclusions and limitations of this study, as well as prospects for further research.

## Materials and Methods

### Framework of the Multidimensional Poverty Index

To measure poverty from a multivariate perspective, we adopt the economic well-being approach and propose a composite measure called the MPI. Following the framework proposed by Alkire and Santos (2010, 2013), methodology related to the computation of the index proposed by Alkire and Foster (2011a, b), and guided by the discussion on multidimensional poverty dimensions presented by Callander et al. (2012), the MPI comprises three dimensions—health, education, and standard of living. The first two dimensions, i.e., health and education, do not have any subdimensions. The third dimension comprises three subdimensions: material deprivation, housing problems, and environment. The MPI framework and the chosen indicators are presented in Table 1.

**Table 1:** Conceptualisation of the MPI

Dimension	Subdimension (index)	Indicators
Health (MPI-H) (1 out of 3)	–	Reporting bad or very bad health conditions (PH010) Unmet need for medical examination or treatment because it was not affordable, there was a waiting list or it was too far to travel/no means of transportation (PH040 and PH050) Unmet need for medical examination or treatment because it was not affordable, there was a waiting list or it was too far to travel/no means of transportation (PH060 and PH070)
Education (MPI-E)	–	Educational attainment: a person Who is more than 24 years old and does not have at least an upper secondary education Who is between the ages 16 and 24 and who finished no more than lower secondary education and is not involved in further education (based on early school leaver definition) (PE010 and PE040)
Standard of living (MPI-L)	Material deprivation (Material Deprivation Index—MDI) (3 out of 9)	HH050—household without ability to keep home adequately warm Households in arrears on HS010/HS011—mortgage or rent payments HS020/HS021—utility bills HS060—lack of capacity to face unexpected financial expenses HS050—(lack of) capacity in a household to afford a meal with meat, chicken, fish (or vegetarian equivalent) every second day HS040—(lack of) capacity in a household to afford paying for 1 week annual holiday away from home Household cannot afford HS070—a telephone (including a mobile phone) HS090—a computer HS100—a washing machine HS110—a car
	Housing problems (Multidimensional Poverty in Housing Index—MPHoI) (2 out of 5)	Crowding index >2 (number of household members divided by HH030—number of rooms available to the household) Problems with dwelling HH040—leaking roof, damp walls/floors/foundation, or rotting window frames or floor HS160—too dark, insufficient light HH080/HH081—without bath or shower for sole use in dwelling HH090/HH091—without indoor flushing toilet for sole use of household
	Environment (Multidimensional Poverty in Environment Index—MPEnI) (2 out of 3)	Household experiences HS170—noise from neighbours or from the street HS180—pollution, grime or other environmental problems HS190—crime violence or vandalism in the area

Although we followed the framework proposed by Alkire and Santos (2010, 2013), it must be noted that the indicators chosen are different because the area of application is considerably different. Alkire and Santos (2010, 2013) computed their index for developing countries, whereas the MPI is computed for the developed countries from the EU. It implies that not only the level but also the nature of the poverty differ. For example, in the approach of Alkire and Santos, indicators related to the availability of electricity or drinking water were included because they describe important issues for developing countries. However, indicators on arrears on mortgage, rent payments or utility bills, among others, were not used because they were neither substantial nor meaningful if applied to the developing countries.

Additionally, contrary to the approach of Alkire and Santos (2010, 2013), we opt for calculating and presenting not only the fully aggregated MPI but also indexes for all three conceptualized dimensions of poverty, namely, the Poverty in Health Index (MPI-H), the Poverty in Education Index (MPI-E), and the Poverty in Standard of Living Index (MPI-L). In this decision, we follow the reasoning of Ravallion (2011, p. 237), who noticed that to prioritise policies for fighting poverty in a given country (or other geographic area), it is necessary to look at the country’s attainments in various dimensions rather than focus on its performance with respect to a single composite index. He also adds that ‘such an approach does not deny that poverty is multidimensional’. Rather it says that ‘forming a single (uni-dimensional) index may not be particularly useful for sound development of policy making’.

Then, to satisfy the requirement that the poverty measure should also enable the ability to assess the intensity of the poverty experienced, we also express the MPI, the MPI-L, the MPI-E, and the MPI-H as products of two measures: the headcount ratio (i.e., H_MPI-H_, H_MPI-E_, and H_MPI-L_) and the intensity of poverty (i.e., A_MPI-H_, A_MPI-E_, and A_MPI-L_). The headcount ratio is a classic measure of poverty that is used, for example, by the Eurostat in the form of the percentage of people below the poverty line, thereby indicating the proportion or incidence of people who are poor. The intensity of poverty is also called the breadth of poverty and relates to the average deprivation score of poor people.

### Data

To populate the MPI framework, we used data from the EU-SILC 2011. As seen in Table 1, the health dimension is measured with three indicators, the education dimension with one indicator, and the standard of living dimension with 17 indicators. It must be noted, however, that while the structure of the first two dimensions is simple, the structure of the standard of living dimension is more complex. It comprises the Material Deprivation Index (MDI), measured with nine indicators, the Multidimensional Poverty in Housing Index (MPHoI), measured with five indicators, and the Multidimensional Poverty in Environment Index (MPEnI), measured with three indicators.^3^

The majority of the chosen indicators relate to households, i.e., they come from the household questionnaire. The only exceptions are the indicator for educational attainment and the indicators for health dimension, namely, PH010: general health; PH020: suffering from any chronic (long-standing) illness or condition; and PH030: limitation in activities because of health problems, all of which are from the personal questionnaire. Although the unit of poverty analysis is not commonly agreed upon (see, for example, Alkire and Santos 2013), we adopt an individual level approach, which implies that we measure poverty among individuals. This means that with respect to the standard of living dimension, in which all indicators measure poverty among households, we assume that if a household is deprived, it implies that all its members are also deprived.

This approach is often applied not only when poverty is measured based on the Alkire and Foster methodology (Alkire and Foster 2011a, b), as is in our case, but also by the Eurostat. Namely, the AROPE rate, despite being measured with household-level indicators on material deprivation, among others, refers to the number of people—not households—at risk of poverty or social exclusion. This household-individual complication results from the fact that in a measurement of poverty with the standard of living dimension included among other dimensions, such as health or education, it happens quite often that the individual-level data (health and education related) and household-level data (related to standard of living) are aggregated into a single measure. In such a case, Alkire and Foster (2011a) suggest that people are identified as poor depending upon the achievements of all household members, which is exactly the strategy we applied.^4^

Because one of our aims is to present the geographical distribution of poverty with respect to the degree of urbanisation, we used the *degurba* variable from the EU-SILC 2011 database. Using it for each of the EU countries, we distinguish three types of areas (EC 2010a, b):

1. Densely populated area (cities/large urban area)—a contiguous set of local areas, each of which has a density >500 inhabitants per square kilometre and where the total population for the set is at least 50,000 inhabitants.
2. Intermediately populated area (towns and suburbs/small urban area)—a contiguous set of local areas not belonging to a densely populated area, each of which has a density >100 inhabitants per square kilometre, and either with a total population for the set of at least 50,000 inhabitants or adjacent to a densely populated area.
3. Sparsely populated area (rural areas)—a contiguous set of local areas belonging neither to a densely populated area nor to an intermediately populated area.

Because in the EU SILC 2011 data for Ireland are not publically available and because two EU countries—the Netherlands and Slovenia—do not provide information on the degree of urbanisation, the poverty estimates are computed for 24 EU countries. Then, because not all categories of degree of urbanisation occur in Estonia, Latvia, Lithuania, and Malta, in these cases the intermediate level of urbanisation is merged with level 1 for Estonia, Latvia, Lithuania, and with level 3 for Malta. Therefore, regional poverty estimates are computed for 69 EU within-country areas.

In other words, for each EU country, four estimates are presented: (1) at the country level, (2) for large urban areas, (3) for small urban areas, and (4) for rural areas. The last three are computed conditional on the degree of urbanisation, which implies that after taking an arithmetic average with the weights corresponding to the share of population living in areas that are different with respect to the degree of urbanisation, the country-level estimate is obtainable.

The measurement of poverty conducted with respect to the degree of urbanisation raises the issue of sample size. In our study, the sample sizes related to each type of degree of urbanisation within each country are for the most part above 1,000. The three exceptions are the intermediately populated areas in Romania, the sparsely populated areas in Belgium and the intermediately populated areas in Bulgaria, for which the sample sizes are 203, 557 and 974, respectively.

### Calculation of the Multidimensional Poverty Index

#### The Sub-Index MPI-H

The sub-index MPI-H is directly computed from the indicators derived from the EU-SILC according to the following rule: A person is considered multidimensionally poor with respect to health if she is deprived in at least two out of three health indicators (if her deprivation score is ≥2/3).

#### The Sub-Index MPI-E

As regards the education dimension, a person is defined as poor with respect to education if she is deprived with respect to the educational attainment indicator described in Table 1. It must also be noted that because there is only one education indicator calculated differently with respect to age, the MPI-E is exactly the same as the H_MPI-E_, and the A_MPI-E_ is always equal 100 %.

#### The Sub-Index MPI-L

As stated previously, the structure of the MPI-L is more complex. Not only does the MPI-L comprise lower-level sub-indexes (the MDI, the MPHoI, and the MPEnI), but in addition, all of them are multidimensional in nature. All lower-level indexes are directly computed from the indicators derived from the EU–SILC (all of them referring to households) according to the following rules:

- As regards the MDI, a household is defined as materially deprived if it is deprived of at least three out of nine indicators (if its deprivation score is at least equal to 1/3).
- As regards the MPHoI, a household is defined as deprived with respect to housing if it is deprived of at least two out of five housing indicators (if its deprivation score is higher than 1/3).
- As regards the MPEnI, a household is defined as deprived with respect to the environment if it is deprived of at least two out of three environment indicators (if its deprivation score is higher than 1/3).

Then, the household-level estimate of poverty in standard of living is assigned to all household members.

The MPI-L is computed as a composite of its three lower-level sub-indexes. Each of these sub-indexes is associated with equal weight (i.e., 1/3). Thus, a person is defined as multidimensionally poor with respect to living standards if she is deprived of at least one of three living standard sub-indexes (if her deprivation score is at least equal to 1/3).

It is very important to note that although all poverty thresholds presented above are established following suggestions formulated by Alkire and Santos (2010, 2013), their influence on the poverty estimates is illustrated in Sect. 3.5.

#### The Multidimensional Poverty Index

Although the MPI has a three-dimensional structure, in its computation, the subdimensional level is also taken into account. More precisely, to give more importance to subdimensions, the formula that attempts to define a multidimensionally poor person comprises lower-level sub-indexes. Therefore, a person is defined as multidimensionally poor if her overall deprivation score is >1/3. Accordingly, the deprivation score for each individual with respect to multidimensional poverty is computed taking into consideration the weighting scheme presented in Table 2 and according to the formula:

$$\begin{aligned} depriv_{score} & = \frac{1}{9} \times GH + \frac{1}{9} \times MD + \frac{1}{9} \times DD + \frac{1}{6} \times EA + \frac{1}{6} \times MDI \\ & \quad + \frac{1}{6} \times MPHoI + \frac{1}{6} \times MPEnI, \\ \end{aligned}$$

where GH is General health, MD is Unmet medical need, DD is Unmet dental need, EA is educational attainment.

**Table 2:** Weighting scheme of the MPI

Dimension	Weight	Sub-dimension	Weight
Health	2/6	General health	1/9 = 2/6 × 1/3
		Suffering from chronic illness	1/9
		Suffering from activity limitations due to health	1/9
Education	1/6	Educational attainment	1/6
Standard of living	3/6	MDI	1/6 = 1/3 × 3/6
		MPHoI	1/6
		MPEnI	1/6

Although the existing research for the most part supports the equal weighting scheme for the poverty dimensions, especially when the Alkire–Foster methodology (Alkire and Foster 2011a, b) is applied, having consulted with experts in the field,^5^ we assigned them arbitrarily. That is, we decided to weigh the standard of living dimension as 1/2, the health dimension as 1/3, and the educational dimension as 1/6. To determine the influence of such a weighting scheme on the results, following the approach of Alkire and Santos (Alkire and Santos 2010), we present different scenario solutions (see Sect. 3.5). It should be noted, however, that the optimal way to assess the robustness of the composite indicator to the normative assumptions made during the construction process is through uncertainty and sensitivity analyses (Paruolo et al. 2013; Saisana et al. 2005). In our case, however, due to large sample sizes and computational complexity, it was not feasible to conduct them.^6^

## Results

We start the presentation of the results with the multidimensional poverty estimates. To this end, we first present the MPI and then supplement it with the presentation of the classical headcount ratio (H_MPI_) and intensity of poverty (A_MPI_). All measures are presented both for the EU countries and for the within-country areas defined by the degree of urbanisation for each EU country (to indicate the existing differences in the levels of poverty within a country). The same strategy applies to three sub-indexes of the MPI. Each of them is presented (i.e., the MPI-H, MPI-E, and MPI-L) together with the classical headcount ratio (i.e., the H_MPI-H_, H_MPI-E_, and H_MPI-L_) and the intensity of poverty (i.e., the A_MPI-H_, A_MPI-E_, and A_MPI-L_), both for countries and for the areas defined by the degree of urbanisation for each EU country. Then, we present several scenarios according to different poverty thresholds and weighting schemes to better visualise the influence of the normative methodological choices on the results.

### Poverty in the European Union

**Fig. 1:**
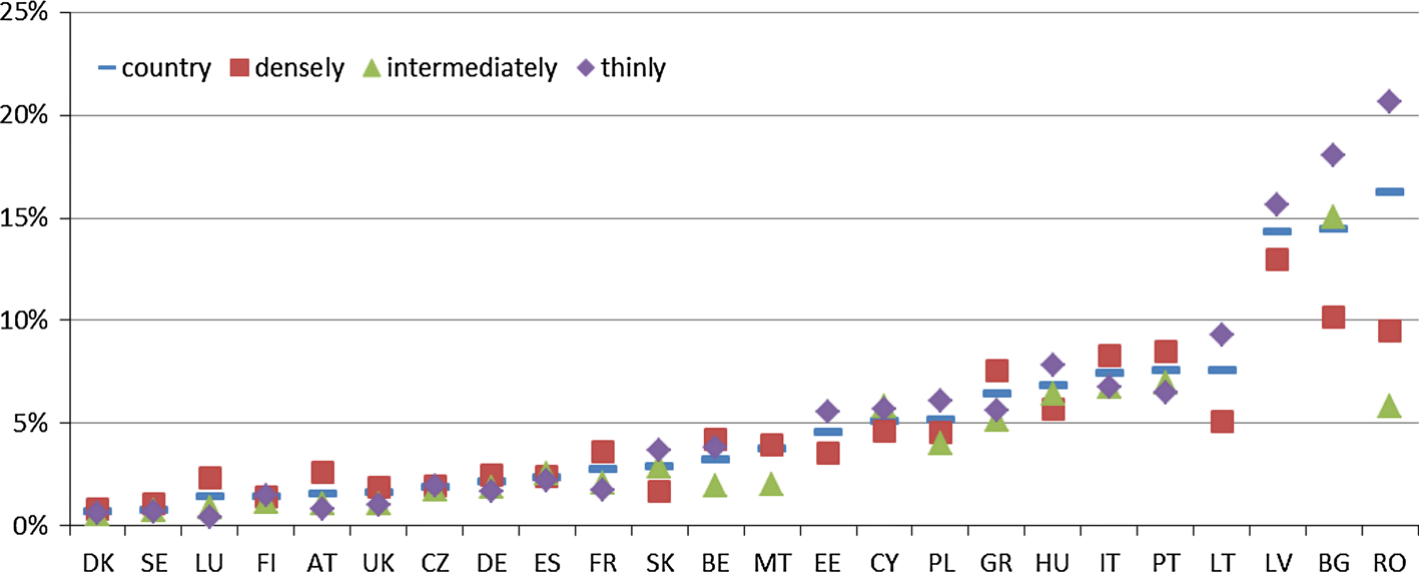
Multidimensional poverty in the EU—the MPI estimates at the country level and by degree of urbanization in 2011 *Note* Country = estimate at a country level, densely = densely populated area, intermediately = intermediately populated area; thinly = thinly populated area

While taking into consideration country-level estimates of the MPI (see Fig. 1; Table 7 in the “Appendix”), we can see that the best-scoring countries (with the lowest poverty level) are Denmark and Sweden with an MPI below 1 %. They are followed by Luxembourg, Finland, Austria, the United Kingdom, and the Czech Republic—all with an MPI below 2 %.

A moderate level of poverty (with an MPI ranging between 2 and 5 %) is observed in Germany, Spain, France, Slovakia, Belgium, Malta, and Estonia. A worse situation is noted in the southern European countries excluding Spain and Malta, namely, in Cyprus, Greece, Italy, and Portugal, and in the three Central and Eastern European (CEE) countries (Poland, Hungary, and Lithuania), all with MPI scores ranging between 5 and 10 %. The worst situation with respect to poverty measured by the MPI is present in Latvia, Bulgaria, and Romania, with MPI scores between 14 and 17 %.

The countries with a relatively high poverty level also demonstrate considerable dissimilarity among the areas that are differentiated with respect to the degree of urbanisation (Fig. 1; Table 6 in the “Appendix”). Considerably higher poverty in thinly populated areas was observed in the CEE countries, with the highest differences observed in Romania and Bulgaria. In the case of Romania, the difference in poverty rates between the intermediately populated and the sparsely populated areas amounts to 14.8 percentage points (pp.). Regarding Bulgaria, the difference between the densely populated and the sparsely populated areas amounts to 7.9 pp.

On the other end of the scale, there are countries that are almost entirely homogenous with respect to poverty estimates. This group includes Sweden, Spain, Finland, Denmark, and the Czech Republic, with maximum differences of <0.5 pp. In the overall analysis, there are also identified countries with very low differences, such as Germany, the United Kingdom, Italy, Malta, Austria, France, and Luxembourg, with maximum differences of approximately 2 pp.

In general, there is observed a positive relationship (r = 0.793) between the stratification level and the MPI, implying that the poorer a country is, the greater the differences between within-country areas defined differently with respect to the degree of urbanisation. However, as mentioned above, the pattern of differences varies. In the poor and moderately poor countries (from lowest-scoring Romania to Estonia), the worst situations with respect to poverty are observed in sparsely populated areas, with the exception of three southern European countries, Greece, Italy, and Portugal, where the worst situations are in densely populated areas. On the other hand, in this group of countries, the best situation, with respect to poverty, is detected in densely populated areas, with the exception of Romania, Poland, Malta, and Belgium, where the worst situations occur in intermediately populated areas. Conversely, in the best-scoring countries, poverty is, generally, relatively higher in densely populated areas than in other areas. This situation applies to Luxembourg, Austria, France, and Belgium. The only exception is Slovakia, where the worst situation related to poverty is noted in sparsely populated areas, though the differences are not considerable.

Such results may be related to the immigration issue. It is known that well-developed countries, especially those with an open labour market, are attractive for immigrants, who, in turn, settle in large cities, i.e., densely populated areas. Such behaviour seems natural, as in large cities, the opportunities for a better quality of life are more numerous. Nevertheless, immigrants, often poor, constitute small and closed local communities, bringing about an increase in social and material inequality.

**Fig. 2:**
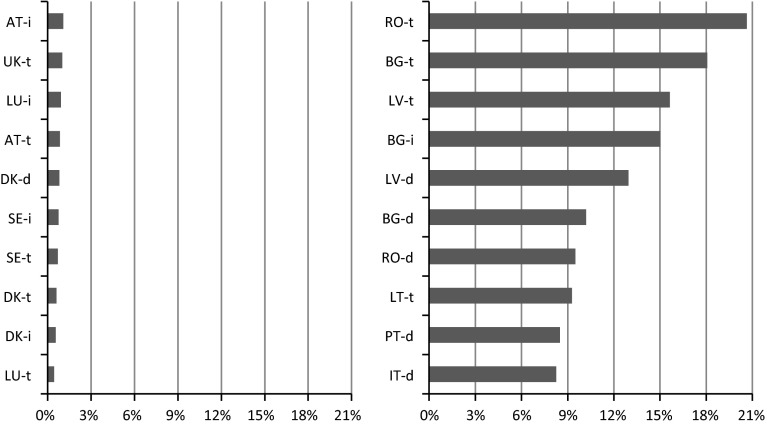
Ten best-scoring (*left*) and ten lowest-scoring (*right*) EU areas with respect to the MPI, defined by degree of urbanisation *Note* d = densely populated area, i = intermediately populated area, t = thinly populated area

The above findings indicate that poverty-related country rankings may be misleading because there is considerable stratification^7^ of poverty with respect to the degree of urbanisation. To better visualise this issue, we present the ten highest-scoring and ten lowest-scoring areas defined by the degree of urbanisation in Fig. 2. It is worth noting that among the best scoring, there are no Finnish areas, although the country is ranked as the fourth best. Among the worst scoring areas, there are no intermediately populated areas in Romania, though this is the most poverty stricken country. On the other hand, there are densely populated areas in Italy, though the country ranks as the sixth worst.

**Fig. 3:**
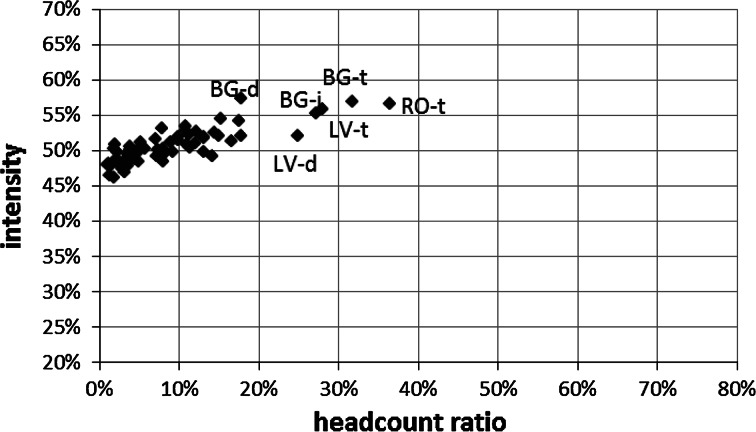
The correspondence between the multidimensional poverty head count ratio (H_MPI_) and multidimensional poverty intensity (A_MPI_) in the EU *Note* d = densely populated area, i = intermediately populated area, t = thinly populated area

In addition to the positive relationship between the stratification level and the MPI, there is also a positive relationship (r = 0.836) between the level of poverty measured by the classical headcount ratio (H_MPI_) and the intensity of poverty (A_MPI_) (Fig. 3). This relationship suggests that in areas where there is a significantly larger number of poor people, these people are also more likely to be poor in more dimensions.

### Poverty in Health in the European Union

**Fig. 4:**
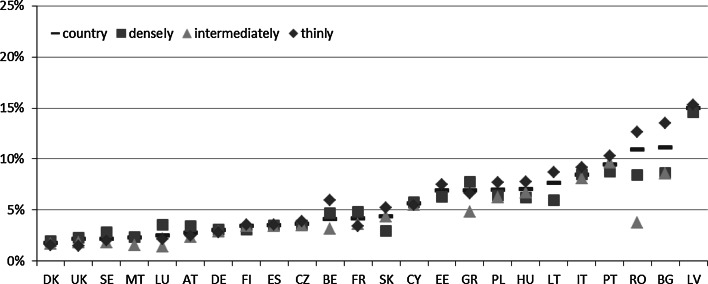
Poverty in health in the EU—the MPI-H estimates at the country level and by degree of urbanization in 2011 *Note* Country = estimate at a country level, densely = densely populated area, intermediately = intermediately populated area, thinly = thinly populated area

While taking into consideration country-level estimates of the MPI-H (see Fig. 4; Table 7 in the “Appendix”), we can observe that the best-scoring country (with the lowest health related poverty level) is Denmark with an MPI-H below 2 %. Then, the United Kingdom, Sweden, Malta, Luxembourg, and Austria follow—all with an MPI-H below 3 %.

A moderate level of poverty (with an MPI-H ranging between 3 and 5 %) is observed in Germany, Finland, Spain, the Czech Republic, Belgium, France, and Slovakia. A relatively bad situation exists in the southern European countries excluding Spain and Malta, namely, in Cyprus, Greece, Italy and Portugal, and in the four CEE countries (Poland, Hungary, Estonia, and Lithuania), all with MPI-H scores ranging from 5 to 10 %. The worst situation with respect to poverty in health is found in Romania, Bulgaria, and Latvia, with an MPI-H ranging from 10 to 15 %.

The countries with a relatively high health poverty level also demonstrate considerable stratification among the areas differentiated according to the degree of urbanisation (Fig. 4; Table 7 in the “Appendix”). Considerably higher poverty in thinly populated areas is observed in the CEE countries and Belgium. The highest differences are observed in Romania and Bulgaria—the difference in health poverty rates between the intermediately populated and the sparsely populated areas amounts to 8.9 and 5.0 pp., respectively. On the other end of the scale, there are countries that are almost entirely homogenous with respect to the health poverty estimates. This group includes Malta, Latvia, Finland, the Czech Republic, Denmark, Germany, Cyprus, and Spain, with maximum differences of <1 pp.

In general, there is observed a moderate positive relationship (r = 0.475) between the stratification level and the MPI-H, implying that, again, the poorer with respect to health a country is, the greater the differences between areas differing with respect to the degree of urbanisation.

In addition to the positive relationship between the stratification level and the MPI-H, there is also a relatively strong positive relationship (r = 0.607) between the level of poverty in health measured by the classical headcount ratio (H_MPI-H_) and the intensity of poverty in health (A_MPI-H_). This relationship suggests that in areas in which there are a significantly large number of poor with respect to health people, these people are also more likely to be poor in more health related dimensions.

**Fig. 5:**
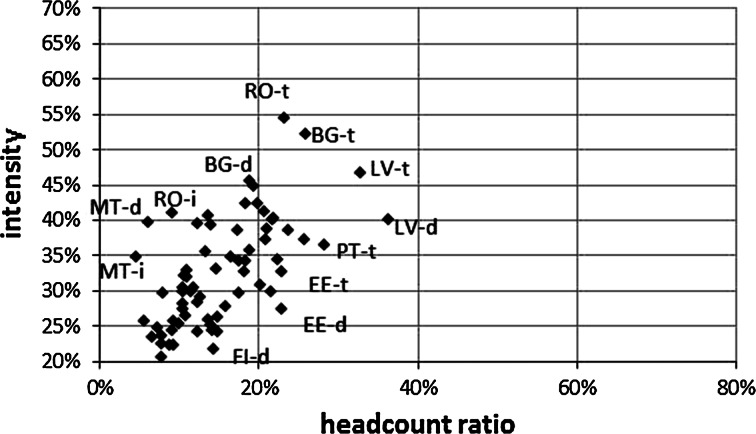
The correspondence between poverty in health head count ratio (H_MPI-H_) and poverty in health intensity (A_MPI-H_) in the EU *Note* d = densely populated area; i = intermediately populated area; t = thinly populated area

There are, however, exceptions to this regularity such as the Estonian regions, where, despite the relatively high number of poor people, the intensity of being poor, namely, the number of health indicators with respect to which a person is poor, is relatively low. On the contrary, there are areas, such as the Maltese regions or the intermediately populated Romanian areas,^8^ in which a considerably high intensity of poverty in health corresponds to a relatively low number of poor in this respect (Fig. 5).

### Poverty in Education in the European Union

**Fig. 6:**
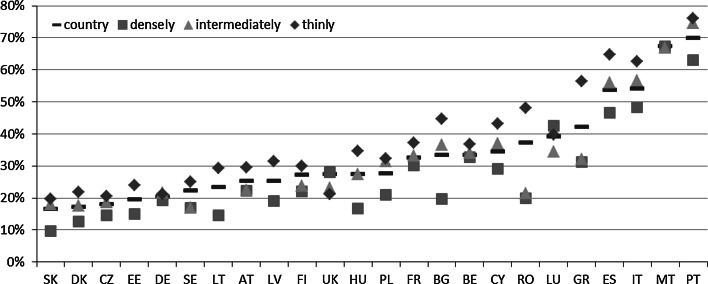
Poverty in education in the EU—the MPI-E estimates at the country level and by degree of urbanization in 2011 *Note* Country = estimate at a country level, densely = densely populated area, intermediately = intermediately populated area, thinly = thinly populated area

While taking into consideration country-level estimates of the MPI-E (see Fig. 6; Table 7 in the “Appendix”), we can observe that the best-scoring countries (with the lowest poverty level) are Slovakia, Denmark, the Czech Republic, and Estonia, all with an MPI-E below 20 %. They are followed by Germany, Sweden, Lithuania, Austria, and Latvia—all with an MPI-E between 20 and 25 %.

A moderate level of poverty in education (with an MPI-E ranging from 26 to 30 %) is observed in Finland, the United Kingdom, Hungary, and Poland. A relatively bad situation exists in France, Bulgaria, Belgium, Cyprus, Romania, and Luxembourg, where the MPI-E ranges from 30 to 40 %. The worst situation with respect to poverty in education is undoubtedly depicted in the southern European countries excluding Cyprus, namely, in Greece, Spain, Italy, Malta, and Portugal, all with MPI-E scores above 40 % and in Malta and Portugal even exceeding 60 %.

Differences among the areas differentiated according to the degree of urbanisation observed with respect to poverty in education is considerably higher than with respect to other poverty dimensions and with respect to poverty itself (Fig. 6 and Table 7 in the “Appendix”). First, higher poverty in thinly populated areas is observed in nearly all countries. The only exceptions are Luxembourg and the United Kingdom, where the poorest with respect to education are people from densely populated areas, and Malta and Germany, where almost no stratification is spotted. Second, the highest differences with respect to the poverty in education are observed in Romania, Greece, and Bulgaria—the difference in education poverty rates between the densely populated and the sparsely populated areas amounts to 28.2, 25.1 and 24.9 pp, respectively. High differences are also spotted in Hungary and Spain. This again relates to the differences in education poverty rates between the densely populated and the sparsely populated areas. The difference amounts to 18.0 pp. On the other end of the scale, there are countries that are almost entirely homogenous with respect to education poverty estimates. These are, as mentioned above, Malta and Germany (0.4 and 2.2 pp. difference between densely and sparsely populated areas, respectively) but also Belgium, the Czech Republic, and the United Kingdom, with observed differences below 7 pp.

In the case of poverty in education, there is observed only a weak positive relationship (r = 0.161) between the stratification level and the MPI-E. This implies that, again, it is not necessarily the case that the poorer with respect to education a country is, the greater are the differences between within-country areas differing with respect to the degree of urbanisation.

### Poverty in Living Standards in the European Union

**Fig. 7:**
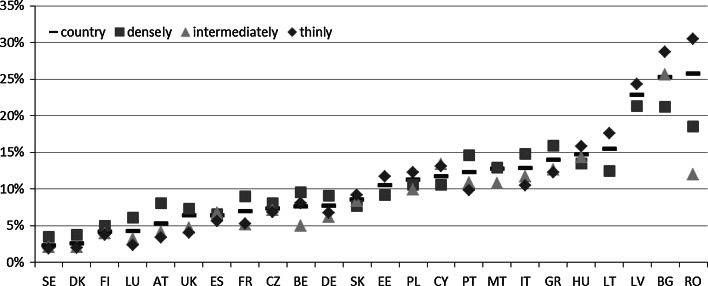
Poverty in living standards in the EU—the MPI-L estimates at the country level and by degree of urbanization in 2011 *Note* Country = estimate at a country level, densely = densely populated area, intermediately = intermediately populated area, thinly = thinly populated area

While taking into consideration country-level estimates of the MPI-L (see Fig. 7; Table 7 in the “Appendix”), we can observe that the best-scoring countries (with the lowest poverty level related to living standards) are Sweden, Denmark, Finland, and Luxembourg, all with an MPI-L below 5 %. These countries are followed by Austria, the United Kingdom, Spain, France, the Czech Republic, Belgium, Germany, and Slovakia—all with an MPI-L between 5 and 10 %. A moderate level of poverty (MPI-L scores between 10 and 15 %) is observed in the CEE countries, namely, Estonia, Poland, and Hungary and in the southern European countries such as Cyprus, Portugal, Malta, Italy, and Greece. The worst situation with respect to poverty in living standards is undoubtedly depicted in Latvia, Bulgaria, and Romania, where the MPI-L amounts to 22.9, 25.3 and 25.8, respectively.

Regarding the differences among the areas differentiated according to the degree of urbanisation, two patterns can be observed (Fig. 7; Table 7 in the “Appendix”). There is a group of countries in which considerably higher poverty in densely populated areas is observed. This group includes the more affluent countries (with respect to poverty in living standards) but also the southern European countries. However, in the CEE countries, considerably higher poverty in thinly populated areas is observed. It is also worth noting that in medium-scoring Belgium, moderately low-scoring Malta and the lowest-scoring Romania, the lowest poverty is observed in the intermediately populated areas.

Undeniably, the highest difference between the areas of different degrees of urbanisation is observed in Romania. It relates to thinly and intermediately populated areas and amounts to 18.5 pp.^9^ Bulgaria and Lithuania follow, but the observed differences in these cases are considerably lower and amount to 7.5 and 5.1 pp., respectively. Slightly lower differences are observed in Portugal, Austria, Belgium, and Italy, all ranging from 4.3 to 4.8 pp. On the other end of the scale, there are countries that are almost entirely homogenous with respect to living standard poverty estimates, with differences between 1 and 2 pp. This group includes northern European countries, such as Finland, Sweden, and Denmark, the CEE countries, such as the Czech Republic and Slovakia, and one southern European country, Spain.

In general, our results indicate that a relatively strong positive relationship (r = 0.644) between the stratification level and the MPI-L is observed. This implies that, again, the poorer with respect to the living standard a country is, the greater are the differences between within-country areas differing with respect to the degree of urbanisation.

**Fig. 8:**
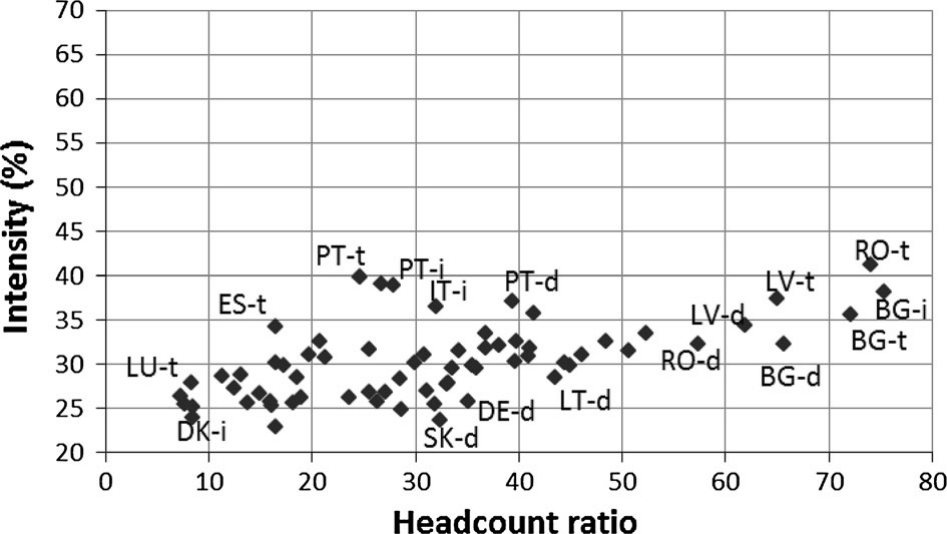
The correspondence between poverty in living standard head count ratio (H_MPI-L_) and poverty in living standard intensity (A_MPI-L_) in the EU *Note* d = Densely populated area, i = intermediately populated area, t = thinly populated area

In addition to the positive relationship between the stratification level and the MPI-L, there is also a relatively strong positive relationship (r = 0.595) between the level of poverty in living standards measured by the classical headcount ratio (H_MPI-L_) and the intensity of poverty in living standards (A_MPI-L_). This relationship suggests that in areas where there are a significantly large number of poor with respect to living standards, these people are also more likely to be poor in more dimensions related to living standards (Fig. 8).

### Scenario Solutions

**Fig. 9:**
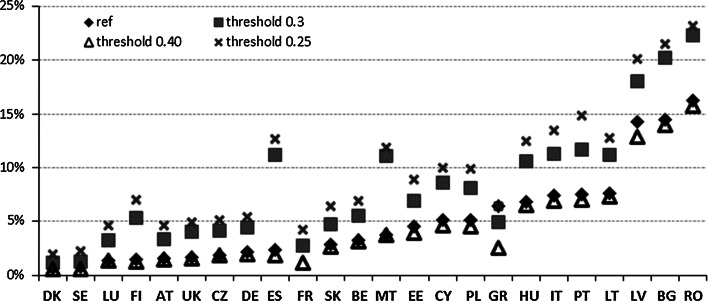
Scenario solutions for the MPI—different poverty thresholds

We present the simulated MPI country estimates providing different poverty thresholds and different weighting schemes. For the poverty threshold, we test three scenarios—poverty threshold set at 25 % (scenario 1a), at 30 % (scenario 2a), and at 40 % (scenario 3a) of the deprivation score (Fig. 9). Upon analysing the results, we find that Scenario 3a in general corresponds to the reference scenario. Scenarios 1a and 2a, on the other hand, differ from the baseline scenario, with results for scenario 1a being the most divergent. Then, we can also see that the differences between simulated MPI scores and the reference ones are the lowest for the most affluent countries, namely, Denmark and Sweden, and in general increase for more disadvantageous countries. This finding, despite being likely related to the natural lower variability of the low scores recorded for the most affluent countries, is in line with the results of the uncertainty analysis recorded for other composite indicators (see, for example, Annoni et al. 2012; Saisana and Weziak-Bialowolska 2013, among others), in which it is often indicated that the best (but often also the worst) scoring countries are characterised by the lowest variability.

The findings imply that though the difference between the examined poverty thresholds from scenarios 1a and 3a and the reference threshold is similar, the influence on the results is stronger in the case of scenario 1a. Therefore, the exercise with different scenarios indicates that not only does the level of poverty depend nonlinearly on the poverty threshold but also that changes in the poverty rate are considerable, especially when the threshold is decreasing.

With regard to the simulation of the weighting schemes, we test two scenarios—equal weights at the dimension level (scenario 1b) and equal weights at the subdimension level (scenario 2b). Due to changes in the weighting scheme, in scenario 1b compared to the reference scenario, the influence of the educational dimension on the MPI is larger, followed by the health dimension, while the living standard dimension exhibits a decreasing impact on the MPI. In scenario 2b, the health and living standard dimensions are treated equally and exhibit equal influence on the MPI, while the influence of the educational dimension decreases with respect to the reference scenario.

**Fig. 10:**
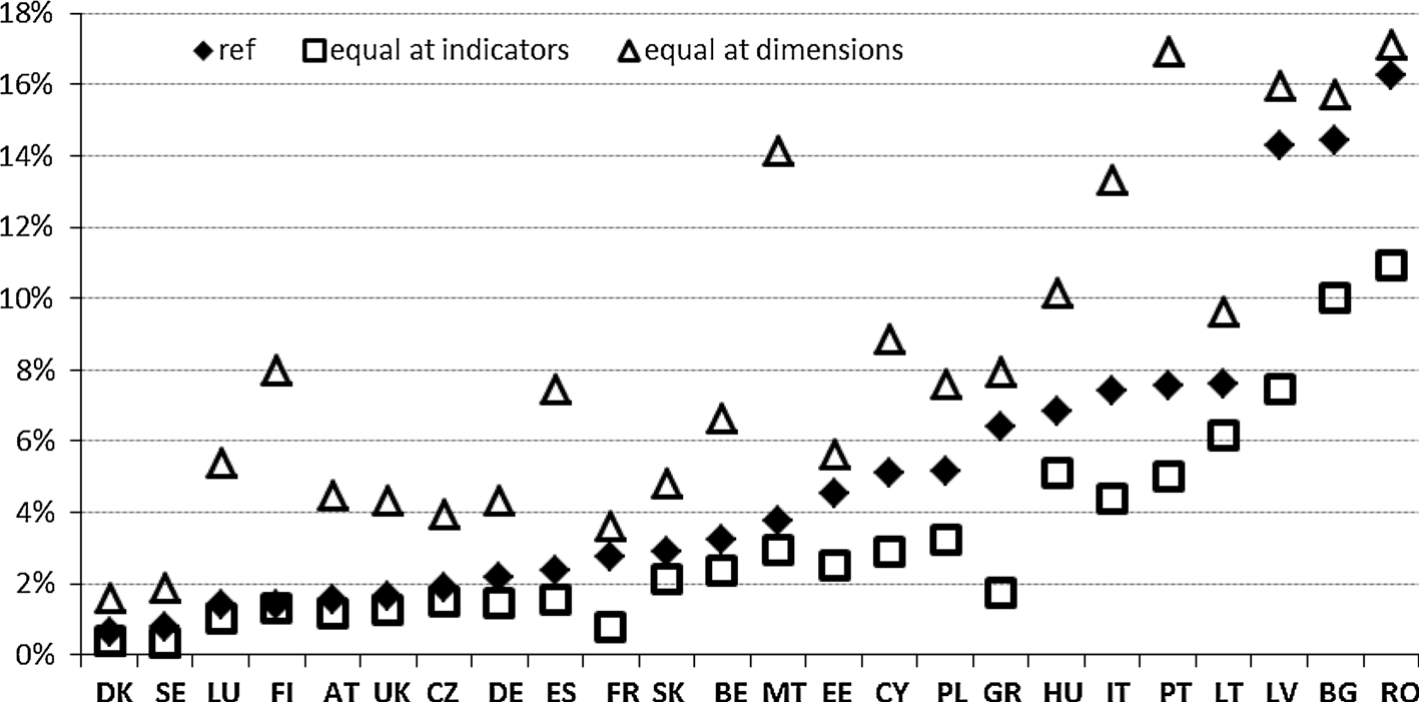
Scenario solutions for the MPI—different weighting scheme

As can be observed, in the scenario with an equal weighting scheme at the dimension level, the results are considerably different. This directly results from the increase in importance of the least important dimension in the reference scenario, namely, education. Indirectly, this result derives also from the fact that the dimensions are populated with a considerably different number of indicators (Fig. 10).

The scenario solutions indicate that as long as there is not a single universal and commonly accepted solution to measuring poverty in a multidimensional way, following the methodology proposed by Alkire and Foster (2011a, b), the outcomes of the proposed measurements are likely to be volatile. This volatility results from the normativity linked to the methodological choices that are unavoidable. The possible solution to this problem is to calculate the poverty estimates with error terms representing the uncertainty related to these choices. Our exercise with the scenario solutions provides considerable contribution with this respect.

### MPI and AROPE

As one can argue about the purpose of proposing an additional poverty indicator for the EU, in this section we demonstrate that, although the two measures seem to be similar, the MPI gives additional information compared to the AROPE rate. We recall that the AROPE rate is a headcount ratio, implying that from the construction point of view, it corresponds directly to the headcount ratios H_MPI_, H_MPI-H_, H_MPI-E_, and H_MPI-L_. Therefore, to illustrate associations between the AROPE and the MPI, we correlate the AROPE rate:

1. with the MPI and its three sub-indexes, i.e., MPI-H, MPI-E, and MPI-L,
2. with the poverty headcount ratios and poverty intensities in each of the poverty dimensions and with respect to multidimensional poverty, i.e., H_MPI-H_, H_MPI-E_, H_MPI-L_, H_MPI_, A_MPI-H_, A_MPI-E_, A_MPI-L_, and A_MPI_.

The analysis is performed for the poverty estimates at the country level and by the degree of urbanisation. The Pearson’s correlation coefficients measuring the relationship between the AROPE and the poverty measures are presented in Table 3, whereas in Table 4, the Pearson’s correlation coefficients between the stratification with respect to our poverty measures and the stratification with respect to the AROPE are displayed.

**Table 3:** Pearson’s correlation coefficients between the MPI and the AROPE rate

Multidimensional poverty		Health		Education		Living standards	
Index	AROPE	Index	AROPE	Index	AROPE	Index	AROPE
Analysis accounting for different with respect to degree of population areas							
MPI	0.880	MPI-H	0.754	MPI-E	0.275	MPI-L	0.870
H_MPI_	0.877	H_MPI-H_	0.583	H_MPI-E_	0.275	H_MPI-L_	0.822
A_MPI_	0.773	A_MPI-H_	0.830	A_MPI-E_	n.a.	A_MPI-L_	0.710
Country level analysis							
MPI	0.909	MPI-H	0.824	MPI-E	0.119	MPI-L	0.901
H_MPI_	0.902	H_MPI-H_	0.679	H_MPI-E_	0.119	H_MPI-L_	0.875
A_MPI_	0.836	A_MPI-H_	0.845	A_MPI-E_	n.a.	A_MPI-L_	0.716

**Table 4:** Pearson’s correlation coefficients between the stratification with respect to the MPI and the stratification with respect to the AROPE

Multidimensional poverty		Health		Education		Living standards	
Index	AROPE	Index	AROPE	Index	AROPE	Index	AROPE
MPI	0.772	MPI-H	0.756	MPI-E	0.549	MPI-L	0.691

First, we can see that with respect to multidimensional poverty, the AROPE rate is highly correlated with all the MPI, H_MPI_, and A_MPI_. Second, it can be noticed that the relationship between the multidimensional poverty measures and the AROPE rate is in general stronger for the estimates at the country level. The only exception to this regularity is that the estimate for the education dimension (MPI-E), which is considerably lower compared to coefficients related to other dimensions, is higher when the degree of urbanisation is taken into account. Third, of the dimensions of multidimensional poverty, the living standard dimension is correlated to the largest degree with the AROPE rate, both at the country level and by the degree of urbanisation. This regularity is observed not only for the headcount ratio H_MPI-L_ but also for the MPI-L. Finally, as regards the MPI-H, measuring the health dimension both at the country level and by degree of urbanisation, the strongest relationship with the AROPE rate is observed for the poverty in health intensity measure A_MPI-H_, whereas the headcount ratio H_MPI-H_ is correlated the weakest.

These results indicate the moderate similarity between the MPI and the AROPE rate. However, it must be noted that especially while reported for the within-country areas, the two measures inform about the level of poverty in a different way. Nevertheless, positive correlation between the AROPE rate and the MPI, especially when analysed at the subnational level (computed with respect to degree of urbanisation), implies that in the regions where people are deprived with respect to income, labour or materially (as the AROPE is conceptualised), people are also likely to be deprived with respect to living standards in general and/or health and/or education.

Then, because both measures comprise a material deprivation component of very similar conceptualisation, it is not surprising that at the dimension level, the highest correlation is observed between the AROPE rate and the MPI-L. We recall that the MPI-L measures poverty in living standards for which one of the components is material deprivation. Next, the substantially weak correlation between the AROPE rate and the MPI-E implies that the MPI comprises a conceptually different component than the AROPE rate and, thus, enriches the picture of poverty measurement.

**Fig. 11:**
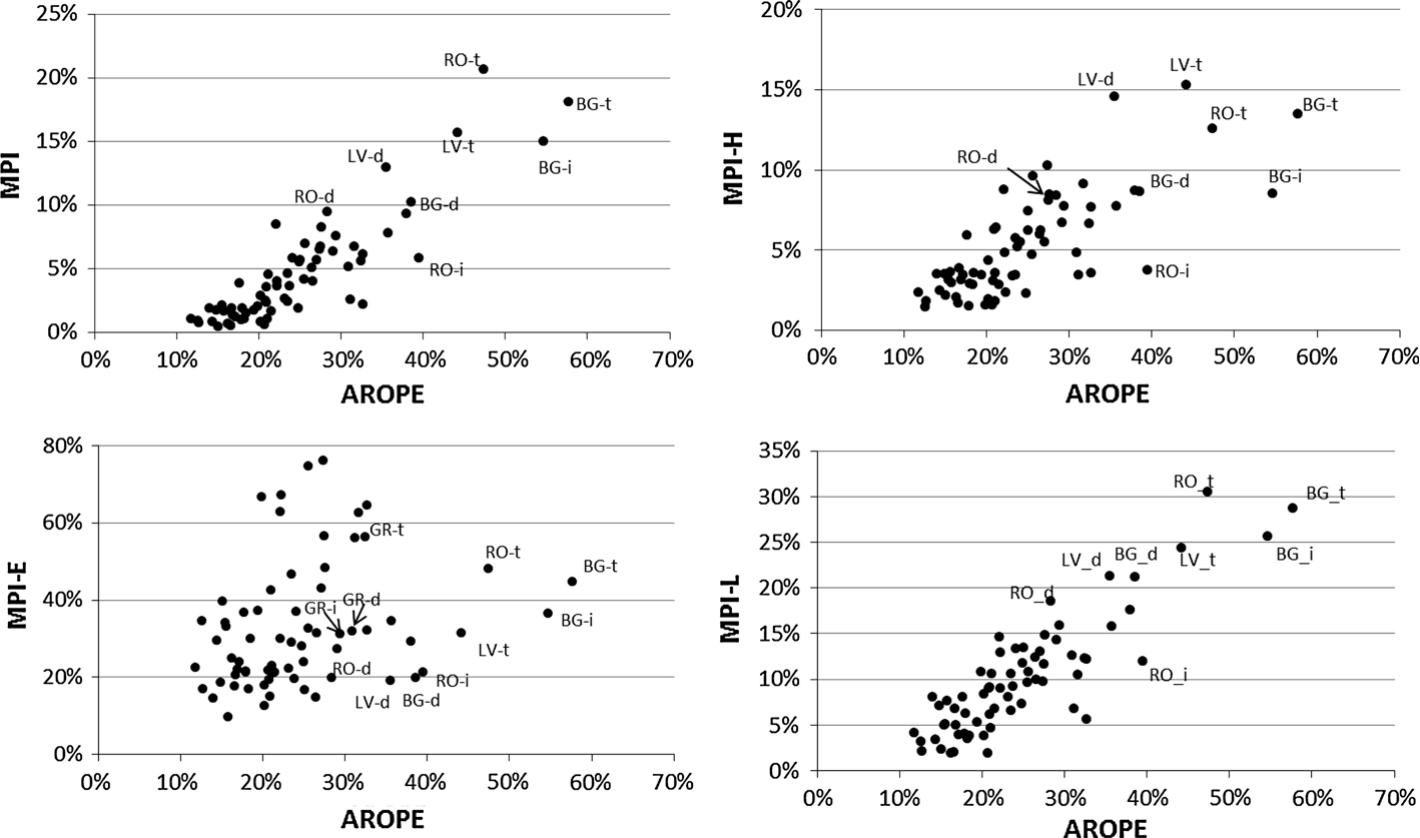
Association for the within-country areas between the MPI, MPI-H, MPI-E, MPI-L and the AROPE (countries with the highest stratification are distinguished) *Note* d = densely populated area, i = intermediately populated area, t = thinly populated area

As regards the association between the stratification with respect to the AROPE and the MPI, MPI-H, MPI-E, and MPI-L, as can be concluded from the analysis of Table 4 (i.e., high correlations between the AROPE and the MPI, MPI-H, MPI-E, and MPI-L) and Fig. 11, the most stratified countries with respect to the AROPE are nearly always also the most stratified ones with respect to other measures. These are the areas in Romania, Bulgaria, and Latvia. The only exception to this regularity is observed while analysing the association between the AROPE and the MPI-E. In this case, the highest differences between the areas of different degrees of urbanisation, according to the MPI-E, are recorded for Romania, Greece, and Bulgaria and also Spain, Hungary, and Cyprus. This finding again confirms that due to incorporation into the MPI framework, the educational dimension the picture of the geographical poverty distribution recorded using the MPI is deepened.

## Conclusions

In this paper, we attempted to measure poverty in the EU countries and in the within-country areas defined by the degree of urbanisation. We first adapted the conceptual model of this phenomenon proposed by Alkire and Santos (2010, 2013) to the area of application, namely, the EU. We decided that the MPI comprises three dimensions—health, education and standard of living [including (1) material deprivation, (2) housing problems and (3) environment components], implying that, contrary to the AROPE rate, it focuses only on nonincome and outcome-related deprivation. After taking into consideration the availability of the data, we summarised and fitted the dimensions of poverty into a composite indicator, namely, the MPI.

Our results indicate that the level of poverty understood mainly as the economic well-being in the EU ranges from 0.7 to 16.3 %, with Denmark and Sweden having unequivocally the lowest share of poor people and Latvia, Bulgaria, and Romania having the largest proportion of poor people. We also demonstrate that there is a positive relationship between the levels of geographical stratification in poverty and magnitude of poverty according to all poverty measures. This positive relationship implies that there are countries in which there is no geographical stratification with respect to poverty (such as Denmark, Sweden, Spain, Finland, and the Czech Republic) and countries, usually poor ones, such as Romania, Bulgaria, and Lithuania, where considerable geographical stratification with respect to poverty occurs. In general, in countries with a high and moderately high number of poor, the worst situation with respect to poverty is observed in sparsely populated areas, and the best situation occurs in densely populated areas with the exception of three southern European countries, Greece, Italy, and Portugal, where the worst situations are in densely populated areas. On the other hand, in the most affluent countries, poverty is relatively higher in the densely populated areas compared to the sparsely populated areas.

The MPI has several useful properties. First, it simultaneously evaluated poverty with respect to the fraction of people who live in multidimensional poverty (the headcount ratio) and multidimensional poverty intensity, i.e., the number of poverty dimensions for which poor people are deprived. Second, it provides information about the level and intensity of poverty with respect to each of its dimensions, i.e., health, education, and standards of living. Third, it informs about the absolute magnitude of poverty experienced by the Europeans in a given country and provides information about the relative standing of the country. Finally, the MPI indicates the geographical distribution of poverty within a country with respect to the degree of urbanisation.

The developed measure of poverty (MPI) has certain limitations. First, although research on poverty has developed rapidly in recent years, it has failed to guide us in establishing aggregation weights or a commonly accepted poverty threshold. This failure led us to apply a particular weighting scheme and particular poverty thresholds. To assess their influence on the final MPI scores, we presented several scenario solutions illustrating the possible variability of the MPI scores. Unfortunately, we were not able to perform a full-scope uncertainty analysis of the MPI. Such an analysis could have demonstrated the simultaneous influence of the imposed weights and of the determined poverty thresholds on the MPI scores.

Our study has clear implications for future research. First, this study calls for computing the MPI for a longer time period and considering all EU countries. Second, such computations should be conducted separately for (1) rural areas, (2) towns and suburbs/small urban areas and (3) cities or large urban areas, i.e., the categories of the degree of urbanisation classification because not only are these concepts often used in the political discourse, but they are also often characterised by different levels and determinants of poverty. Further, an in-depth empirical research, most likely employing individual-level data and multi-level modelling, is necessary to test not only the usefulness of the MPI but also to investigate institutional determinants of multidimensional poverty. Finally, it would be worthwhile to compare the results obtained using the composite indicator approach used by us with the results obtained using the latent class approach, such as the one proposed by Dewilde (2008) and Dewilde and Vranken (2005). To this end, instead of the ECHP data originally used by the authors, the EU-SILC 2011 data should be used, and the percentages of the poor could be compared.

## Appendix

See Tables 5, 6 and 7.

**Table 5:** Percentage of people experiencing a deprivation with respect to the indicators of the MPI

Country	Area	Sample size	PH010 (%)	PH020 (%)	PH030 (%)	% of people poor with respect of educational attainment	% of people having housing problem	% of people materially deprived	% of people experiencing problems with environment
AT	AT_1	3,923	9.5	0.8	2.0	22.3	4.0	15.0	15.5
AT	AT_2	3,013	8.3	0.4	1.2	22.5	3.0	5.5	9.1
AT	AT_3	4,539	9.2	0.1	0.8	29.6	2.6	6.0	5.1
BE	BE_1	5,983	9.9	2.0	3.6	32.8	6.0	15.4	17.4
BE	BE_2	4,924	8.7	0.8	2.1	34.1	3.7	7.9	7.4
BE	BE_3	557	13.9	2.0	4.9	36.8	5.7	16.3	6.0
BG	BG_1	6,562	9.3	8.3	9.5	19.8	22.2	50.8	23.1
BG	BG_2	974	10.6	7.3	8.6	36.7	27.6	67.1	12.9
BG	BG_3	7,789	14.2	11.4	12.2	44.7	33.3	68.8	8.9
CY	CY_1	5,192	7.3	4.8	9.2	29.1	3.6	23.2	15.6
CY	CY_2	1,283	6.1	4.8	7.0	37.0	7.6	29.1	17.3
CY	CY_3	3,025	8.1	2.9	5.4	43.1	7.3	27.8	14.5
CZ	CZ_1	5,527	11.5	1.1	1.8	14.5	3.0	15.4	20.3
CZ	CZ_2	4,319	12.7	1.0	1.1	18.7	3.5	16.4	12.9
CZ	CZ_3	7,766	13.2	1.2	1.2	20.5	3.3	17.5	9.5
DE	DE_1	11,613	7.9	1.9	2.3	19.4	2.4	13.3	25.4
DE	DE_2	8,930	8.2	1.7	2.0	21.6	1.8	11.1	14.2
DE	DE_3	3,677	8.6	1.6	1.9	21.3	2.1	14.7	13.3
DK	DK_1	1,777	6.8	0.5	1.9	12.7	2.0	3.4	13.2
DK	DK_2	2,175	6.4	0.7	1.4	17.7	1.1	2.8	5.3
DK	DK_3	1,370	6.5	0.7	1.1	21.9	2.0	1.7	4.6
EE	EE_1	3,363	15.2	8.3	7.9	14.9	3.1	23.2	12.6
EE	EE_3	7,808	17.1	6.4	7.7	24.0	20.1	20.9	5.8
ES	ES_1	13,888	7.3	0.6	3.9	46.7	1.7	12.0	10.4
ES	ES_2	5,909	7.1	0.6	3.8	56.1	2.5	12.4	9.4
ES	ES_3	9,153	7.9	0.4	3.3	64.7	2.2	12.6	3.8
FI	FI_1	2,132	5.3	4.1	6.4	22.0	1.8	9.5	10.3
FI	FI_2	1,285	7.3	4.6	4.9	23.9	1.8	8.1	7.0
FI	FI_3	5,934	8.3	4.7	4.0	30.0	2.8	7.9	4.2
FR	FR_1	9,046	8.6	3.1	5.7	30.1	4.1	14.4	17.6
FR	FR_2	8,033	8.8	1.7	3.9	33.2	3.2	9.6	6.7
FR	FR_3	4,228	9.0	1.4	2.9	37.2	5.6	11.4	3.8
GR	GR_1	3,939	7.5	9.6	8.8	31.3	3.5	28.4	32.2
GR	GR_2	1,290	6.5	5.4	5.4	32.0	2.6	27.2	20.5
GR	GR_3	7,412	11.2	6.0	5.6	56.3	5.0	28.4	8.2
HU	HU_1	7,222	14.5	2.8	5.0	16.8	8.8	37.5	12.9
HU	HU_2	5,207	15.2	2.5	4.3	27.5	8.5	41.7	8.6
HU	HU_3	12,206	17.6	2.5	4.2	34.7	11.0	45.3	5.9
IT	IT_1	15,193	12.3	6.2	9.0	48.3	6.1	21.9	25.2
IT	IT_2	16,045	13.0	5.5	9.9	56.7	5.3	22.4	10.3
IT	IT_3	9,258	16.2	6.2	9.4	62.6	5.4	22.0	3.3
LT	LT_1	4,448	14.2	4.4	4.3	14.6	7.2	31.9	16.9
LT	LT_3	6,582	21.8	2.1	3.4	29.4	27.6	38.8	5.2
LU	LU_1	4,832	10.1	0.9	1.8	42.5	3.5	6.4	13.1
LU	LU_2	3,835	4.9	0.4	0.6	34.5	1.6	3.7	7.7
LU	LU_3	2,784	8.5	0.3	0.4	39.8	2.5	3.2	3.5
LV	LV_1	6,181	15.6	17.5	22.6	19.0	13.1	48.3	22.8
LV	LV_3	7,322	17.6	14.0	18.3	31.4	30.4	52.3	11.0
MT	MT_1	8,438	4.2	1.1	1.5	67.4	1.7	16.2	28.5
MT	MT_2	1,016	4.3	0.3	0.3	66.9	2.3	15.9	19.1
PL	PL_1	10,592	13.6	8.3	5.6	21.0	6.2	24.4	14.2
PL	PL_2	4,877	13.6	7.7	4.7	31.5	6.8	26.8	6.1
PL	PL_3	14,952	16.2	7.8	5.0	32.2	11.0	30.7	5.4
PT	PT_1	4,215	14.6	1.4	9.2	63.0	5.1	23.3	21.2
PT	PT_2	4,234	19.1	1.4	9.7	74.7	3.8	20.6	7.9
PT	PT_3	4,040	22.8	1.3	8.0	76.1	5.4	18.5	4.6
RO	RO_1	5,231	8.9	11.0	10.7	19.9	9.4	41.6	29.9
RO	RO_2	203	6.2	3.1	2.5	21.4	9.9	38.3	4.8
RO	RO_3	10,477	10.0	12.7	13.4	48.1	56.0	52.1	12.4
SE	SE_1	1,355	6.0	1.1	4.7	16.9	1.3	3.7	9.8
SE	SE_2	1,061	5.0	0.6	3.3	17.0	1.5	2.0	5.2
SE	SE_3	4,301	5.8	0.9	3.3	25.0	0.8	3.1	4.0
SK	SK_1	3,371	10.1	1.7	1.6	9.8	1.2	17.9	18.6
SK	SK_2	4,295	13.2	2.7	2.2	18.0	3.1	21.2	12.4
SK	SK_3	5,773	15.5	2.1	1.8	19.7	4.5	26.7	8.9
UK	UK_1	9,035	5.8	1.3	1.6	28.0	3.8	13.7	15.1
UK	UK_2	3,710	5.6	1.2	1.4	23.1	2.9	9.6	9.1
UK	UK_3	2,159	4.2	1.1	2.1	21.3	5.0	6.5	7.1

**Table 6:** Levels of poverty with respect to the MPI, MPI-H, MPI-E, and MPI-L—estimates by degree of urbanisation

Country	Area	Multidimensional poverty (%)			Poverty in health (%)			Poverty in education (%)	Poverty in living standards (%)		
		H_MPI_	A_MPI_	MPI	H_MPI-H_	A_MPI-H_	MPI-H	MPI-E	H_MPI-L_	A_MPI-L_	MPI-L
AT	AT_1	5.1	51.2	2.6	11.3	29.9	3.4	22.3	28.5	28.4	8.1
AT	AT_2	2.2	49.8	1.1	9.2	25.9	2.4	22.5	15.9	25.7	4.1
AT	AT_3	1.7	48.5	0.8	9.8	25.4	2.5	29.6	12.5	27.2	3.4
BE	BE_1	7.9	53.2	4.2	13.2	35.7	4.7	32.8	30.8	31.1	9.6
BE	BE_2	3.9	50.2	1.9	10.3	30.6	3.2	34.1	16.5	30.1	5.0
BE	BE_3	7.9	48.4	3.8	18.1	32.8	5.9	36.8	25.5	31.7	8.1
BG	BG_1	17.8	57.3	10.2	19.2	44.9	8.6	19.8	65.6	32.3	21.2
BG	BG_2	27.2	55.2	15.0	18.7	45.6	8.5	36.7	72.1	35.6	25.7
BG	BG_3	31.8	56.9	18.1	25.8	52.4	13.5	44.7	75.4	38.1	28.7
CY	CY_1	9.2	49.9	4.6	16.4	35.0	5.8	29.1	35.8	29.5	10.6
CY	CY_2	11.2	52.2	5.9	13.5	40.8	5.5	37.0	44.4	30.1	13.4
CY	CY_3	11.3	50.4	5.7	13.9	39.5	5.5	43.1	41.0	31.9	13.1
CZ	CZ_1	3.8	50.5	1.9	13.5	25.9	3.5	14.5	31.9	25.4	8.1
CZ	CZ_2	3.5	49.6	1.7	13.8	25.3	3.5	18.7	28.6	24.9	7.1
CZ	CZ_3	3.9	49.6	1.9	14.7	26.3	3.9	20.5	25.5	26.8	6.8
DE	DE_1	5.0	50.4	2.5	10.4	30.0	3.1	19.4	35.1	25.8	9.1
DE	DE_2	3.8	49.8	1.9	10.3	28.3	2.9	21.6	23.6	26.3	6.2
DE	DE_3	3.4	48.8	1.7	10.6	26.7	2.8	21.3	26.3	25.8	6.8
DK	DK_1	1.7	46.2	0.8	8.7	22.4	1.9	12.7	16.5	22.9	3.8
DK	DK_2	1.2	48.2	0.6	7.6	22.6	1.7	17.7	8.5	24.0	2.0
DK	DK_3	1.3	46.5	0.6	7.6	20.7	1.6	21.9	7.7	25.5	2.0
EE	EE_1	7.2	49.2	3.5	22.7	27.6	6.3	14.9	33.1	27.7	9.2
EE	EE_3	10.9	50.8	5.5	22.8	32.8	7.5	24.0	36.8	31.9	11.7
ES	ES_1	4.9	48.5	2.4	10.8	32.0	3.5	46.7	21.3	30.7	6.5
ES	ES_2	5.1	50.2	2.6	10.6	32.3	3.4	56.1	20.8	32.5	6.8
ES	ES_3	4.5	48.8	2.2	10.9	32.9	3.6	64.7	16.5	34.2	5.7
FI	FI_1	2.9	47.4	1.4	14.2	21.9	3.1	22.0	18.9	26.2	4.9
FI	FI_2	2.5	48.0	1.2	14.1	24.4	3.4	23.9	14.9	26.8	4.0
FI	FI_3	3.2	46.9	1.5	14.7	24.3	3.6	30.0	13.2	28.8	3.8
FR	FR_1	7.1	51.6	3.6	14.6	33.1	4.8	30.1	29.9	30.1	9.0
FR	FR_2	4.2	49.6	2.1	12.5	29.2	3.6	33.2	17.2	29.8	5.1
FR	FR_3	3.7	48.6	1.8	12.2	28.4	3.5	37.2	18.6	28.5	5.3
GR	GR_1	14.4	52.6	7.6	18.3	42.4	7.8	31.3	50.6	31.5	15.9
GR	GR_2	9.9	52.0	5.1	12.3	39.6	4.9	32.0	40.9	30.8	12.6
GR	GR_3	11.1	50.6	5.6	17.2	38.8	6.7	56.3	36.7	33.5	12.3
HU	HU_1	10.8	52.9	5.7	18.2	34.3	6.3	16.8	44.9	29.9	13.4
HU	HU_2	12.1	52.7	6.4	18.8	35.8	6.7	27.5	46.1	31.0	14.3
HU	HU_3	15.0	52.1	7.8	20.7	37.4	7.7	34.7	48.4	32.6	15.8
IT	IT_1	15.2	54.4	8.3	20.6	41.3	8.5	48.3	41.4	35.7	14.8
IT	IT_2	13.1	51.8	6.8	20.9	38.8	8.1	56.7	32.0	36.5	11.7
IT	IT_3	13.0	51.9	6.8	23.6	38.7	9.1	62.6	26.7	39.1	10.5
LT	LT_1	9.9	51.5	5.1	17.4	34.4	6.0	14.6	43.5	28.5	12.4
LT	LT_3	17.8	52.1	9.3	21.7	40.2	8.7	29.4	52.4	33.5	17.5
LU	LU_1	4.7	49.6	2.3	11.6	30.6	3.6	42.5	19.7	31.1	6.1
LU	LU_2	1.8	50.3	0.9	5.5	25.9	1.4	34.5	11.3	28.6	3.2
LU	LU_3	0.9	47.9	0.4	8.9	24.4	2.2	39.8	8.4	27.9	2.3
LV	LV_1	24.9	52.1	12.9	36.3	40.2	14.6	19.0	61.9	34.5	21.3
LV	LV_3	28.0	55.9	15.6	32.7	46.8	15.3	31.4	65.0	37.5	24.4
MT	MT_1	7.9	50.2	4.0	6.0	39.8	2.4	67.4	39.8	32.5	12.9
MT	MT_2	4.2	49.0	2.0	4.5	34.9	1.6	66.9	34.3	31.6	10.8
PL	PL_1	8.9	51.1	4.5	21.4	29.9	6.4	21.0	35.5	29.9	10.6
PL	PL_2	8.1	49.5	4.0	20.0	31.0	6.2	31.5	33.6	29.6	9.9
PL	PL_3	12.0	51.1	6.1	22.3	34.5	7.7	32.2	38.1	32.1	12.2
PT	PT_1	16.5	51.4	8.5	21.8	40.3	8.8	63.0	39.4	37.1	14.6
PT	PT_2	14.2	49.2	7.0	25.6	37.4	9.6	74.7	27.8	39.0	10.8
PT	PT_3	13.0	49.8	6.5	28.1	36.6	10.3	76.1	24.6	39.8	9.8
RO	RO_1	17.5	54.2	9.5	19.8	42.5	8.4	19.9	57.3	32.3	18.5
RO	RO_2	10.8	53.5	5.8	9.1	41.1	3.7	21.4	39.7	30.3	12.0
RO	RO_3	36.4	56.7	20.6	23.1	54.7	12.6	48.1	74.0	41.2	30.5
SE	SE_1	2.3	48.7	1.1	10.3	27.5	2.8	16.9	13.7	25.6	3.5
SE	SE_2	1.6	48.1	0.7	7.2	24.8	1.8	17.0	8.4	25.2	2.1
SE	SE_3	1.5	48.0	0.7	9.2	22.4	2.1	25.0	7.2	26.4	1.9
SK	SK_1	3.5	47.8	1.7	12.3	24.4	3.0	9.8	32.4	23.7	7.7
SK	SK_2	5.7	50.3	2.9	15.6	27.8	4.4	18.0	31.1	27.0	8.4
SK	SK_3	7.3	50.2	3.7	17.5	29.8	5.2	19.7	33.1	27.9	9.2
UK	UK_1	3.9	49.2	1.9	7.8	29.8	2.3	28.0	27.0	26.9	7.3
UK	UK_2	2.2	48.4	1.1	7.7	23.7	1.8	23.1	18.2	25.6	4.7
UK	UK_3	2.0	50.9	1.0	6.4	23.5	1.5	21.3	16.1	25.4	4.1

**Table 7:** Levels of poverty with respect to the MPI, MPI-H, MPI-E, and MPI-L—the country level estimates

Country	Multidimensional poverty (%)			Poverty in health (%)			Poverty in education (%)	Poverty in living standards (%)		
	H_MPI_	A_MPI_	MPI	H_MPI-H_	A_MPI-H_	MPI-H	MPI-E	H_MPI-L_	A_MPI-L_	MPI-L
AT	3.1	50.3	1.6	10.2	27.3	2.8	25.2	19.1	27.5	5.3
BE	6.2	52.2	3.3	12.2	33.7	4.1	33.5	24.7	30.9	7.6
BG	25.4	56.9	14.4	22.5	49.2	11.1	33.3	70.9	35.6	25.3
CY	10.1	50.4	5.1	15.3	36.9	5.6	34.3	38.6	30.4	11.7
CZ	3.8	49.9	1.9	14.1	25.9	3.6	17.9	28.5	25.8	7.4
DE	4.3	50.0	2.2	10.4	28.9	3.0	20.4	29.6	25.9	7.7
DK	1.4	46.9	0.7	8.0	22.1	1.8	17.1	10.9	23.7	2.6
EE	9.1	50.2	4.5	22.7	30.2	6.9	19.5	35.0	29.9	10.5
ES	4.8	49.0	2.4	10.8	32.3	3.5	53.6	19.9	31.9	6.4
FI	3.0	47.1	1.4	14.5	23.7	3.4	27.1	14.9	27.7	4.1
FR	5.4	50.7	2.8	13.4	31.0	4.2	32.5	23.4	29.8	7.0
GR	12.4	51.8	6.4	17.0	40.5	6.9	42.2	43.3	32.1	13.9
HU	13.0	52.4	6.8	19.5	36.2	7.0	27.4	46.8	31.4	14.7
IT	14.0	53.1	7.4	21.2	39.8	8.4	54.0	35.3	36.4	12.8
LT	14.6	51.9	7.6	19.9	38.1	7.6	23.4	48.8	31.7	15.5
LU	2.8	49.6	1.4	8.9	28.2	2.5	39.1	14.2	30.0	4.2
LV	26.4	54.1	14.3	34.5	43.3	14.9	25.3	63.5	36.0	22.9
MT	7.5	50.1	3.8	5.8	39.4	2.3	67.3	39.2	32.4	12.7
PL	10.2	50.9	5.2	21.6	32.2	7.0	27.6	36.4	30.9	11.2
PT	15.0	50.4	7.5	24.5	38.4	9.4	69.8	32.2	38.1	12.3
RO	29.0	56.1	16.3	21.7	50.4	10.9	37.2	67.3	38.3	25.8
SE	1.6	48.2	0.8	9.1	23.8	2.2	22.3	8.6	26.0	2.2
SK	5.8	49.9	2.9	15.5	28.1	4.4	16.6	32.3	26.5	8.6
UK	3.4	49.1	1.7	7.6	28.0	2.1	27.3	23.8	26.7	6.3

## Footnotes

1 The AROPE is reported (1) at the country level, (2) for different levels of the nomenclature of territorial units for statistics (NUTS) and (3) for differently defined areas with respect to the degree of urbanisation. It consists of three sub-indicators that are derived from the EU-SILC data: (1) a relative component, the at-risk-of poverty rate/monetary poverty (AROPE); (2) a “kind of” absolute component, material deprivation; and (3) an exclusion from the labour market component, severe low work intensity.

2 All of the dimensions correspond to the areas of interest enumerated in the Europe 2020: Europe's growth strategy, launched in the EU in 2010.

3 The percentages of people experiencing deprivation with regard to each of the dimensions are presented in Table 5 in the “Appendix”.

4 There is also a contrary strategy, i.e., to measure poverty taking a household as a basis of the analysis. In such a case, all individual-level variables must be converted into household-level variables. It is usually achieved by taking the value of the head of household as the reference value for the household.

5 Because this paper presents the results of the project commissioned to the European Commission Joint Research Centre (JRC) by the Directorate-General for Regional and Urban Policy (DG REGIO), the consultations were conducted with regional policy experts working in the field of poverty, social exclusion, and living standards and experts in the field of composite indicators.

6 We tried to do it in Matlab, which is the most frequently used software in this respect.

7 In the paper, we understand stratification as the observed difference in poverty level between within-country areas differing with respect to the degree of urbanisation.

8 This finding should, however, be treated with caution due to the small sample size (see Sect. 2.2).

9 Again, this finding should be treated with caution due to the small sample size (see Sect. 2.2).
